# Comparison of Outcomes of Edge‐to‐Edge Mitral Valve Repair Versus Surgical Mitral Valve Repair for Functional Mitral Regurgitation

**DOI:** 10.1002/clc.24313

**Published:** 2024-07-08

**Authors:** Xiqiang Wang, Yanpeng Ma, Zhongwei Liu, Ling Zhu, Junkui Wang, Gongchang Guan, Shuo Pan, Yong Zhang, Yuanyuan Hao

**Affiliations:** ^1^ Key Laboratory of Synthetic and Natural Functional Molecule of the Ministry of Education, Xi'an Key Laboratory of Functional Supramolecular Structure and Materials, College of Chemistry and Materials Science Northwest University Xi'an Shaanxi People's Republic of China; ^2^ Department of Cardiovascular Medicine Shaanxi Provincial People's Hospital Xi'an Shaanxi People's Republic of China; ^3^ Department of Cardiovascular Medicine Xi'an Central Hospital Xi'an Shaanxi People's Republic of China

**Keywords:** edge‐to‐edge mitral valve repair, mitral insufficiency, surgical mitral valve repair

## Abstract

**Aims:**

Patients affected by functional mitral regurgitation represent an increasingly high‐risk population. Edge‐to‐edge mitral valve repair (TEER) has emerged as a promising treatment option for these patients. However, there is limited research on the comparative outcomes of TEER versus surgical mitral valve repair (SMVr). This study seeks to compare the demographics, complications, and outcomes of TEER and SMVr based on a real‐world analysis of the National Inpatient Sample (NIS) database.

**Methods and Results:**

In the NIS database, from the years 2016 to 2018, a total of 6233 and 2524 patients who underwent SMVr and TEER were selected, respectively. The mean ages of the patients were 65.68 years (SMVr) and 78.40 years (TEER) (*p* < 0.01). The mortality rate of patients who received SMVr was similar to that of patients who were treated with TEER (1.7% vs. 1.9%, *p* = 0.603). Patients who underwent SMVr more likely suffered from perioperative complications including cardiogenic shock (2.3% vs. 0.4%, *p* < 0.001), cardiac arrest (1.7% vs. 1.1%, *p* = 0.025), and cerebrovascular infarction (0.9% vs. 0.4%, *p* = 0.013). The average length of hospital stay was longer (8.59 vs. 4.13 days, *p* < 0.001) for SMVr compared to TEER; however, the average cost of treatment was higher ($218 728.25 vs. $215 071.74, *p* = 0.031) for TEER compared to SMVr. Multiple logistic regression analysis showed that SMVr was associated with worse adjusted cardiogenic shock (OR, 7.347 [95% CI, 3.574−15.105]; *p* < 0.01) and acute kidney injury (OR, 2.793 [95% CI, 2.356−3.311]; *p* < 0.01).

**Conclusion:**

Patients who underwent TEER demonstrated a notable decrease in postoperative complications and a shorter hospitalization period when compared to those who underwent SMVr.

## Graphical abstract

There is limited research on the comparative outcomes of TEER versus SMVr for functional mitral regurgitation patients. Our results show that TEER patients had fewer postoperative complications and a shorter hospitalization period compared with SMVr patients.

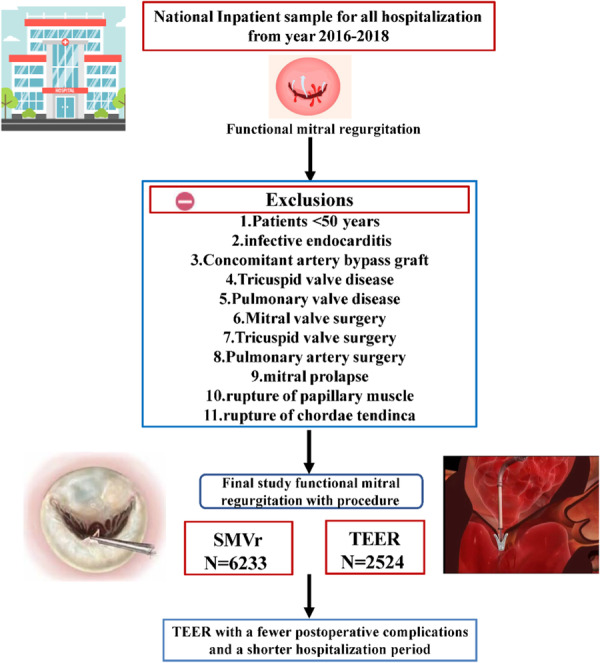

## 1 Introduction

Functional mitral regurgitation (FMR) occurs as a result of left ventricular dilatation and/or papillary muscle dysfunction, rather than any structural abnormalities of the mitral valve (MV) itself. Progressive FMR, which affects one in five FMR patients, is particularly linked to unfavorable outcomes [1]. Patients who have persistent, severe, and symptomatic FMR despite receiving guideline‐directed medical therapy (GDMT) should be referred to a multidisciplinary heart team for evaluation of surgical or transcatheter intervention should be considered [2].

There is currently no conclusive evidence supporting surgical interventions for the treatment of FMR. While mitral annuloplasty, the most commonly utilized technique for surgical mitral valve repair (SMVr), has been shown to reduce SMR and improve symptoms in observational studies [3], its durability and impact on mortality compared to GDMT remain uncertain [4, 5].

The Cardiovascular Outcomes Assessment of the TEER for Heart Failure Patients With Functional Mitral Regurgitation (COAPT) trial recently showed that the addition of M‐TEER to GDMT provided significant benefits in terms of both all‐cause mortality and HF hospitalization in a well‐defined patient cohort [6]. The COAPT trial demonstrated that TEER was beneficial in terms of cumulative HF rehospitalizations, as well as survival rates at both 24 and 36 months of follow‐up [7]. COAPT was the first randomized‐controlled trial to suggest a survival benefit associated with interventional correction of FMR and other studies have also demonstrated the advantages of percutaneous MV repair over other treatment methods [8, 9].

Despite the advantages of percutaneous MV repair, there is a lack of data specifically comparing the outcomes of TEER and SMVr for the treatment of FMR, particularly from randomized‐controlled trials or other high‐quality clinical studies. This study seeks to investigate the burden, outcomes, financial costs, and complications of TMVR and transcatheter mitral valve repair (TMVr) in a real‐world population using the National Inpatient Sample (NIS) database.

## 2 Methods

### 2.1 Study Data

In this study, we used the data from the NIS database from January 2016 to December 2018, which was developed by the Agency from Healthcare Research and Quality of the United States through a federal–state–industry partnership. The NIS database has more than eight million inpatients and represents 20% of all hospital admissions in the United States. Also, it is updated annually, so we can use these data to analyze the disease trend over time. Because the NIS database is publicly available, we did not need to obtain the approval of the institutional review board or informed consent in our clinical study.

### 2.2 Study Design and Data Selection

**Figure 1:**
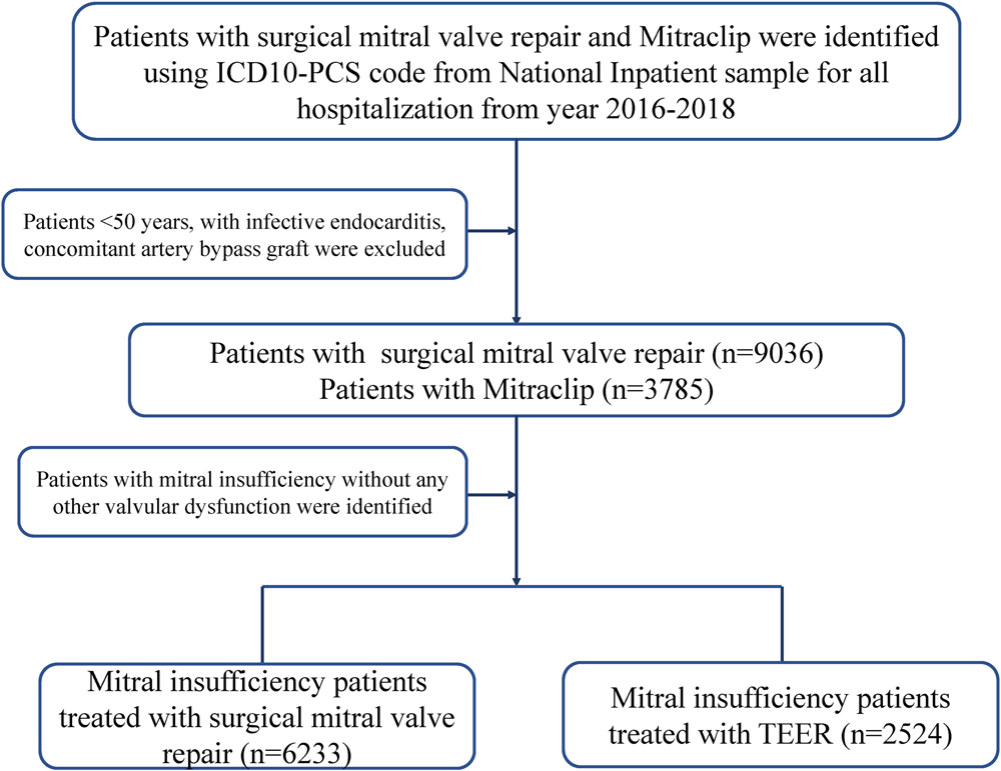
Flowchart of the study cohort. ICD 10‐PCS, International Classification of Diseases, Tenth Revision, Procedure Coding System; TEER, transcatheter edge‐to‐edge mitral valve repair.

The International Classification of Diseases, Tenth Revision, Clinical Modification (ICD‐10‐CM) codes and ICD‐10‐Procedure Coding System (PCS) codes were used to analyze the data. The NIS data from 2016 to 2018 were used in the present study (Table S1). Patients with MV insufficiency and without any other valvular disease were selected using ICD‐10‐CM codes (I340, I051, I341, I342, I050, I351, I061, I350, I060, I352, I062). Patients who underwent SMVr and/or TEER were selected using ICD‐10‐PCS codes (02QG0ZE, 02QG0ZZ) and ICD‐10‐PCS codes (02UG3JZ), respectively. The periprocedural complications post the procedure were identified by the ICD‐10‐CM codes; the detailed ICD‐10‐CM codes are shown in Table S1. Patients who were younger than 50 years of age, patients with infective endocarditis or those who had undergone coronary artery bypass surgery previously, and patients with other valvular diseases were excluded from our study. A flowchart of our patient selection criterion is presented in Figure 1.

### 2.3 Study Outcomes

The primary endpoints of our study were in‐hospital mortality and periprocedural complications. The secondary outcomes of interest were resource use and operative procedure‐related trends over time, such as length of hospital stay, total charges, and the number of SMVr and TEER patients.

### 2.4 Statistical Analysis

The Pearson *χ* ^2^ exact test was used for categorical variables and an independent *t*‐test was used for continuous variables. Categorical variables and continuous variables were presented as frequency and median of standard deviations, respectively. Logistic regression analysis was performed to find the predictors of inpatient mortality, blood transfusion, cardiogenic shock, acute kidney injury, thrombosis due to cardiac prosthetic devices, respiratory failure, and respiratory complications after SMVr and TEER operations using relevant demographic and clinical variables shown in Table 1. For all analyses, a two‐sided *p* value of 0.05 was considered statistically significant. Statistical analyses were performed using SPSS version 25 (IBM, Armonk, NY) and R version 3.5 (version 3.6.3, R Core Team).

**Table 1:** Basic characteristics of patients who underwent SMVr and TEER (2016−2018).

**Characteristics**	**SMVr (*n* = 6233)**	**TEER (*n* = 2524)**	***p* value**
Age, years (mean ± SD)	65.68 ± 8.594	78.40 ± 9.010	< 0.001
Female sex, *n* (%)	2142 (34.4)	1169 (46.3)	< 0.001
Race			0.001
White	4972 (83.6)	1980 (81.8)	
African American	365 (6.1)	172 (7.1)	
Hispanic	282 (4.7)	151 (6.2)	
Asian/Pacific Islander	165 (2.8)	55 (2.3)	
Native American	10 (0.2)	12 (0.5)	
Other races	150 (2.5)	52 (2.1)	
Comorbidities and medical history			
Deficiency anemia, *n* (%)	153 (2.5)	102 (4.0)	< 0.001
Atrial fibrillation, *n* (%)	3511 (56.3)	1516 (60.1)	0.01
Heart failure, *n* (%)	2723 (43.7)	2048 (81.1)	< 0.001
Chronic obstructive pulmonary disease, *n* (%)	746 (12)	597 (23.7)	< 0.001
Coagulopathy, *n* (%)	337 (5.4)	42 (1.7)	< 0.001
Coronary artery disease, *n* (%)	2940 (47.2)	1620 (64.2)	< 0.001
Cerebral hemorrhage, *n* (%)	4 (0.1)	2 (0.1)	0.807
Cerebral infarction, *n* (%)	106 (1.7)	24 (1.0)	0.009
Type 2 diabetes mellitus, *n* (%)	1131 (18.1)	679 (26.9)	< 0.001
Hypertension, *n* (%)	2472 (39.7)	502 (19.9)	< 0.001
Liver disease, *n* (%)	185 (3.0)	89 (3.5)	0.174
Obesity, *n* (%)	910 (14.6)	249 (9.9)	< 0.001
Peripheral vascular disease, *n* (%)	150 (2.4)	67 (2.7)	0.499
Renal failure, *n* (%)	1506 (24.2)	1071 (42.4)	< 0.001
Alcohol use, %	161 (2.6)	21 (0.8)	< 0.001
Tobacco abuse, *n* (%)	124 (2.0)	19 (0.8)	< 0.001
Hyperlipemia, *n* (%)	3066 (49.2)	1415 (56.1)	< 0.001
Acute myocardial infarction, *n* (%)	465 (7.5)	36 (1.4)	< 0.001
Primary payer, *n* (%)			< 0.001
Medicare	3092 (49.7)	2233 (88.6)	
Medicaid	312 (5.0)	46 (1.8)	
Private insurance	2573 (41.3)	206 (8.2)	
Other	249 (4)	285 (11.3)	

Abbreviations: SMVr, surgical mitral valve repair; TEER, transcatheter edge‐to‐edge mitral valve repair.

## 3 Results

### 3.1 Characteristics of Study Participants Selected From the NIS Database

Between January 2016 and December 2018, a total of 6233 patients who underwent SMVr and 2524 patients who underwent TEER were identified (Table 1). Patients who underwent TEER were older compared to those who underwent SMVr (78.40 vs. 65.68 years, *p *< 0.001). Both cohorts included predominantly White patients (83.6% SMVr vs. 81.8% TEER) (Table 1). The use of SMVr and TEER was similar among Hispanic patients (4.7% vs. 6.2%).

### 3.2 Clinical Outcomes in Study Cohort

The in‐hospital mortality rate was similar between SMVr (1.7%) and TEER (1.9%, *p* = 0.603) (Table 2). Cost of care ($215 071.74 ± 179 184.4 vs. $218 728.25 ± 250 279.64, *p* = 0.031) was slightly higher for TEER, but length of stay (8.59 ± 6.61 vs. 4.13 ± 6.45 days, *p* < 0.001) was considerably longer for SMVr (Table 2).

**Table 2:** Clinical outcomes in patients who underwent SMVr and TEER (2016−2018).

Variable	SMVr (*n* = 6233)	TEER (*n* = 2524)	*p* value
In‐hospital mortality, *n* (%)	106 (1.7)	47 (1.9)	0.603
Length of hospital stay, days	8.59 ± 6.61	4.13 ± 6.45	< 0.001
Total charges, $	215 071.74 ± 179 184.4	218 728.25 ± 250 279.64	0.031
Cardiac complications			
Postprocedural cardiogenic shock, *n* (%)	144 (2.3)	9 (0.4)	< 0.001
Postprocedural pericardial complications, *n* (%)	311 (5.0)	65 (2.6)	< 0.001
Cardiac tamponade, *n* (%)	43 (0.7)	11 (0.4)	0.169
Cardiac arrest, *n* (%)	107 (1.7)	27 (1.1)	0.025
IABP, *n* (%)	307 (4.9)	38 (1.5)	< 0.001
ECMO, *n* (%)	33 (0.5)	1 (0.04)	0.001
Hemopericardium, *n* (%)	4 (0.1)	6 (0.2)	0.067
Respiratory complications			
Postprocedural respiratory failure, *n* (%)	246 (3.9)	18 (0.7)	< 0.001
Postprocedural respiratory complications, *n* (%)	340 (5.5)	37 (1.5)	< 0.001
Mechanical ventilation use, *n* (%)	479 (7.7)	104 (4.1)	< 0.001
Other perioperative complications			
Fluid and electrolyte disorders, *n* (%)	2254 (36.2)	383 (15.2)	< 0.001
Postprocedural blood transfusion, *n* (%)	898 (14.4)	97 (3.8)	< 0.001
Postprocedural acute kidney injury, *n* (%)	1073 (17.2)	363 (14.4)	0.01
Postprocedural cerebrovascular infarction, *n* (%)	53 (0.9)	9 (0.4)	0.013
Postprocedural bleeding/hematoma postprocedure, *n* (%)	141 (2.3)	33 (1.3)	0.004
Postprocedural thrombosis due to cardiac prosthetic devices, *n* (%)	15 (0.2)	5 (0.2)	0.706
Postprocedural acute embolism and thrombosis, *n* (%)	72 (1.2)	25 (1.0)	0.505

Abbreviations: ECMO, extracorporeal membrane oxygenation; IABP, intra‐aortic balloon pump; SMVr, surgical mitral valve repair; TEER, transcatheter edge‐to‐edge mitral valve repair.

**Figure 2:**
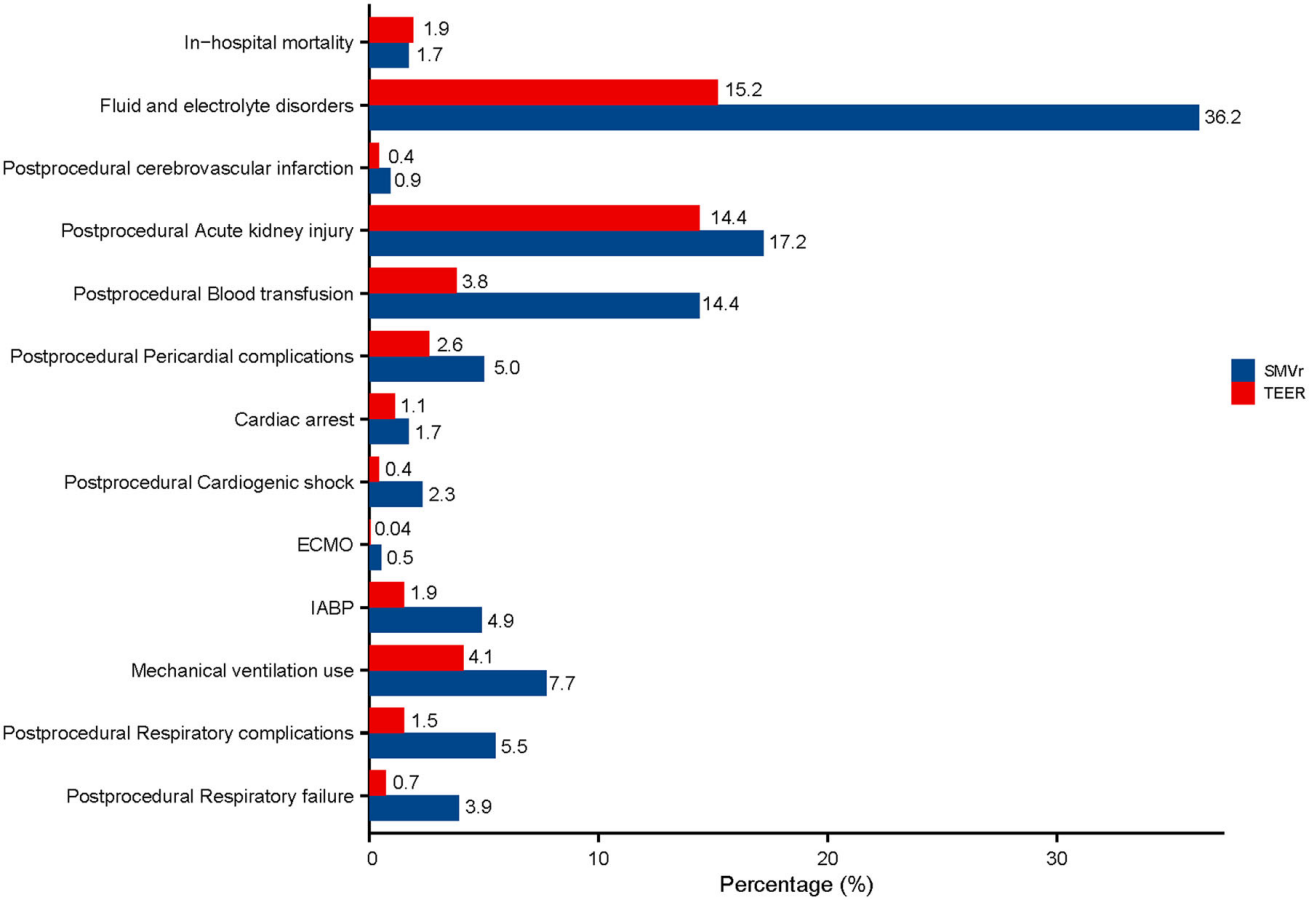
Procedural outcomes in SMVr and TEER in the cohort. ECMO, extracorporeal membrane oxygenation; IABP, intra‐aortic balloon pump; SMVr, surgical mitral valve repair; TEER, transcatheter edge‐to‐edge mitral valve repair.

The patients who underwent SMVr were more likely to suffer from postprocedural cardiogenic shock (2.3% vs. 0.4%, *p* < 0.001), postprocedural pericardial complications (5.0% vs. 2.6%, *p* < 0.001), cardiac arrest (1.7% vs. 1.1%, *p* = 0.025), postprocedural respiratory failure (3.9% vs. 0.7%, *p* < 0.001), postprocedural respiratory complications (5.5% vs. 1.5%, *p* < 0.001), mechanical ventilation use (7.7% vs. 4.1%, *p* < 0.001), fluid and electrolyte disorders (36.2% vs. 15.2%, *p* < 0.001), postprocedural blood transfusion (14.4% vs. 3.8%, *p* < 0.001), postprocedural acute kidney injury (17.2% vs. 14.4%, *p* = 0.01), postprocedural cerebrovascular infarction (0.9% vs. 0.4%, *p* = 0.013), and postprocedural bleeding/hematoma after the procedure (2.3% vs. 1.3%, *p* = 0.004) (Table 2, Figure 2). The usage rate of extracorporeal membrane oxygenation was higher for SMVr (0.5% vs. 0.04%, *p* = 0.005). An intra‐aortic balloon pump was required in 4.9% of patients who underwent SMVr (*p* < 0.001) (Table 2, Figure 2).

### 3.3 Temporal Trends

**Figure 3:**
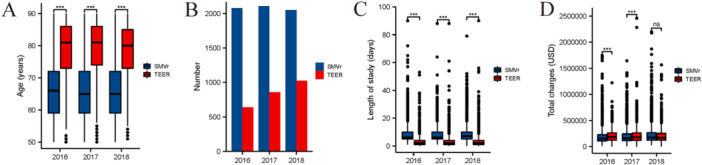
Trends in SMVr and TEER from 2016 to 2018. Trends in age (A), number (B), length of stay (C), and cost of stay (D) of patients undergoing SMVr and TEER from 2016 to 2018 in the NIS database. SMVr, surgical mitral valve repair; TEER, transcatheter edge‐to‐edge mitral valve repair.

Over the study period, the patients in the SMVr group were younger than the patients in the TEER group (Figure 3A). The number of patients in the TEER group increased from 639 to 1024, but the number of patients in the SMVr group remained the same (Figure 3B). The median length of stay remained stable in the SMVR group (8.49−8.69 days) and the TEER group (4.071−4.36 days) (Figure 3C). However, the median cost increased in the SMVr group ($200 679−$232 979) but was the same in the TEER group ($210 943−$215 057) (Figure 3D).

### 3.4 Predictors of Clinical Outcomes

Logistic regression showed that SMVr was associated with a higher occurrence of postprocedural cardiogenic shock (odds ratio [OR], 7.347 [95% CI, 3.574−15.105]; *p* < 0.001). Factors associated with higher postprocedural cardiogenic shock included heart failure (OR, 2.126 [95% CI, 1.405−3.218]; *p* < 0.001), liver disease (OR, 3.905 [95% CI, 2.328−6.564]; *p* < 0.01), and coagulopathy (OR, 2.497 [95% CI, 1.515−4.115]; *p* < 0.001) (Figure S1). Our results also suggested that SMVr was significantly related to higher postprocedural pericardial complications (OR, 1.565 [95% CI, 1.124−2.179]; *p* < 0.01) (Figure S2), postprocedural respiratory failure (OR, 7.501 [95% CI, 4.407−12.768]; *p* = 0.007) (Figure S3), postprocedural respiratory complications (OR, 4.448 [95% CI, 3.024−6.544]; *p* < 0.01) (Figure S4), postprocedural blood transfusion (OR, 7.414 [95% CI, 5.778−9.512]; *p* < 0.001) (Figure S5), postprocedural acute kidney injury (OR, 2.793 [95% CI, 2.356−3.311]; *p* < 0.001) (Figure S6), and postprocedural bleeding/hematoma postprocedure (OR, 2.310 [95% CI, 1.435−3.716]; *p* = 0.001) (Figure S7), but not for postprocedural cerebrovascular infarction (OR, 2.711 [95% CI, 0.997−7.367]; *p* = 0.051) (Figure S8).

## 4 Discussion

In our real‐world study comparing outcomes between TEER and SMVr, we obtained the following main results: (1) the odds of in‐hospital mortality were similar between TEER and SMVr. (2) SMVr was associated with increased periprocedural complications. (3) While SMVr was associated with a longer length of stay compared to TEER, the average cost of treatment was higher for TEER than for SMVr.

Our study contributes valuable data on the outcomes and resource utilization associated with TEER and surgical SMVr in a real‐world population using the NIS database. Although SMVr and TMVr have been compared in a few observational studies and in a subgroup of the randomized Endovascular Valve Edge‐to‐Edge Repair Study (EVEREST), few studies have specifically compared the outcomes of TEER and SMVr for the treatment of SMR. In a study by Kortlandt et al. [10], 365 patients with SMR treated with TMVr were compared with 95 patients who underwent MV surgery. The authors reported similar adjusted survival up to 3 years between the two groups (HR = 0.86; 95% CI, 0.54−1.38; *p* = 0.541). In a subgroup analysis of the EVEREST trial that included 56 patients with SMR, no significant difference was found between TMVr and SMVr for the composite endpoint of death, MV surgery or reoperation, and 3^+^ or 4^+^ SMR at 5 years (28.6% vs. 40.5%; *p* = 0.43) [11].

Our study reveals a steady increase in the utilization of TEER for FMR treatment in the United States between 2015 and 2018, indicating growing use of this procedure at a national level. These findings align with the previous registry data, which also demonstrated a gradual increase in the use of TEER over time in patients with high or prohibitive surgical risk, and established the safety and feasibility of this approach. According to reports, the in‐hospital mortality rate for FMR patients receiving TEER operations ranges from 1.8% to 6% and tends to decrease with experience and technological advancements [12, 13]. Data from TEER‐treated FMR cohorts with a 3% in‐hospital mortality rate were used in our investigation. This was comparable to the SMVr in‐hospital mortality rate of 1.7%.

MV surgery frequently includes several treatment modalities, such as sternotomy or minimally invasive access, further subvalvular therapy, or ventricular remodeling, such as chordal cutting/replacement [14, 15], edge‐to‐edge repair [16], or papillary muscle approximation [17, 18]. Additionally, simultaneous treatments like a tricuspid valve repair, coronary artery bypass graft, pulmonary vein isolation, maze procedures or pulmonary vein isolation, or a left atrial appendage occlusion are commonly carried out. In contrast, in TEER, invasiveness and periprocedural risk are minimized by avoiding the cumulative risk of several operations being carried out simultaneously and adhering to a highly structured workflow. However, concurrent heart diseases can be addressed later using phased therapeutic approaches and low‐risk treatments like catheter ablation (0.15%) [19], elective percutaneous coronary intervention (0.1%) [20], or percutaneous left atrial appendage occlusion (0.3%) [21].

Similar to other studies [6, 22], our study showed that TEER patients were older and had a higher number of comorbidities than non‐TEER patients, suggesting that a higher percentage of patients may be at increased risk for surgery. Although TEER patients, on average, were older than SMVr patients, the SMVr group had more cardiac problems, respiratory issues, and other perioperative complications, showing that SMVr lagged significantly behind TEER in terms of surgical safety.

One of the most dreaded side effects of structural heart surgery is stroke, and it has a significant effect on patient death [23]. Compared to the TEER series, the early stroke rate for current SMVr users was greater at about 4% [24]. Our research also revealed that cerebral infarction in SMVr patients with TEER was greater (1.7% vs. 1.0%) but the stroke rate reported in our study was less than early reported in previous studies [24, 25]. The ideal anticoagulant level during surgical procedures and the optimum anticoagulant regimen during the perioperative period still need to be established, even though these variations may be partially attributed to the learning curve process.

Because of the NIS database's intrinsic flaws, our analysis has several limitations. First, the study's comparison of the heart function before and after the surgery does not include any laboratory or echocardiography data. In the future, more research and clinical trials are required to help formulate evidence‐based guidelines for the management of MV disease. Second, we did not identify the TMVr device, and we did not have information on the resources used and operative procedure‐related trends by region and time distribution. Third, because TMVr is a technique that involves a higher risk in general and may be appropriate for particular clinical and/or anatomical reasons, subgroup data on TMVr in these groups are necessary, and their absence in our study is a significant drawback. Finally, because the NIS database was not created to track patient data longitudinally, the long‐term endpoints could not be assessed in NIS samples.

SMVr has more adverse cardiovascular events and less procedural safety in our study. For the purpose of creating evidence‐based guidelines for the use of catheter management in MV disease, further clinical research and randomized‐controlled trials are required.

## Author Contributions

X.W. and Y.M. conceived the study and wrote the manuscript. Z.L. and L.Z. provided the data, analyzed the data, and revised the manuscript. J.W. revised the manuscript. G.G. and Y.Z. revised the manuscript, reviewed the results and provided guidance for this study. S.P. and Y.H. revised the manuscript and provided guidance for this study.

## Ethics Statement

Because the NIS database is publicly available, we did not need to obtain approval from the institutional review board or informed consent in our clinical study, and our study was carried out in accordance with the Helsinki Declaration guidelines.

## Consent

The authors have nothing to report.

## Conflicts of Interest

The authors declare no conflicts of interest.

## Supporting information

Figure S1. Predictors of postprocedural cardiogenic shock in mitral valve insufficiency patients undergoing SMVr and TEER.

Figure S2. Predictors of postprocedural pericardial complications in mitral valve insufficiency patients undergoing SMVr and TEER.

Figure S3. Predictors of postprocedural respiratory failure in mitral valve insufficiency patients undergoing SMVr and TEER.

Figure S4. Predictors of Postprocedural Respiratory complications in mitral valve insufficiency patients undergoing SMVr and TEER.

Figure S5. Predictors of Postprocedural Blood transfusion in mitral valve insufficiency patients undergoing SMVr and TEER.

Figure S6. Predictors of Postprocedural Acute kidney injury in mitral valve insufficiency patients undergoing SMVr and TEER.

Figure S7. Predictors of Postprocedural Bleeding/hematoma post‐procedure in mitral valve insufficiency patients undergoing SMVr and TEER.

Figure S8. Predictors of Postprocedural cerebrovascular infarction in mitral valve insufficiency patients undergoing SMVr and Mitraclip.

Supporting information.

## Data Availability

All data generated or analyzed during this study are included in this published article.
