# Supplementary figures and images for: Comparison of Outcomes of Edge‐to‐Edge Mitral Valve Repair Versus Surgical Mitral Valve Repair for Functional Mitral Regurgitation
Source: Clin Cardiol. 2024 Jul 8;47(7):e24313. doi: 10.1002/clc.24313 (PMC11228827; doi:10.1002/clc.24313)

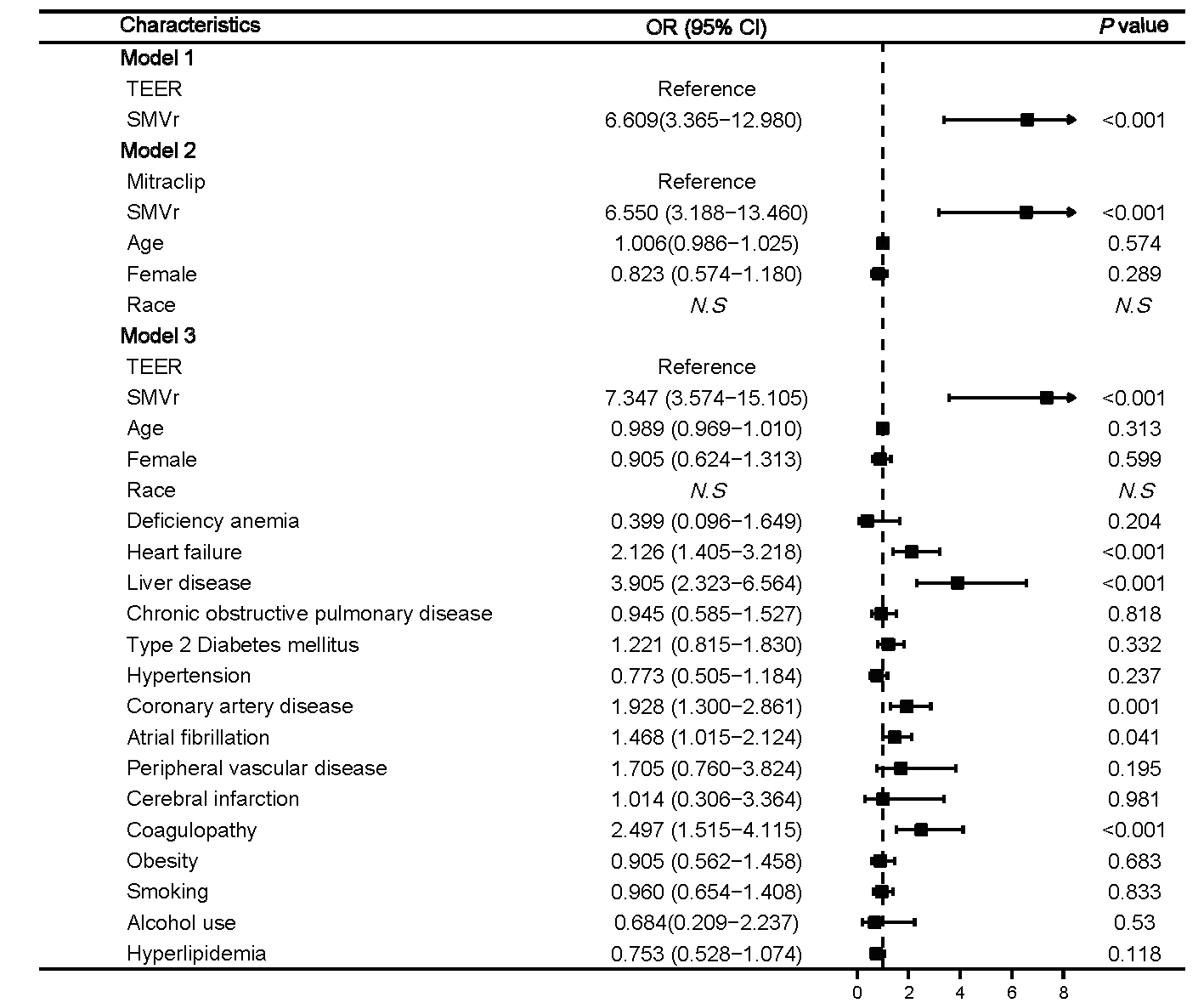

Supplement: Supplementary file 1 — Figure S1. Predictors of postprocedural cardiogenic shock in mitral valve insufficiency patients undergoing SMVr and TEER. [file CLC-47-e24313-s006.tif]

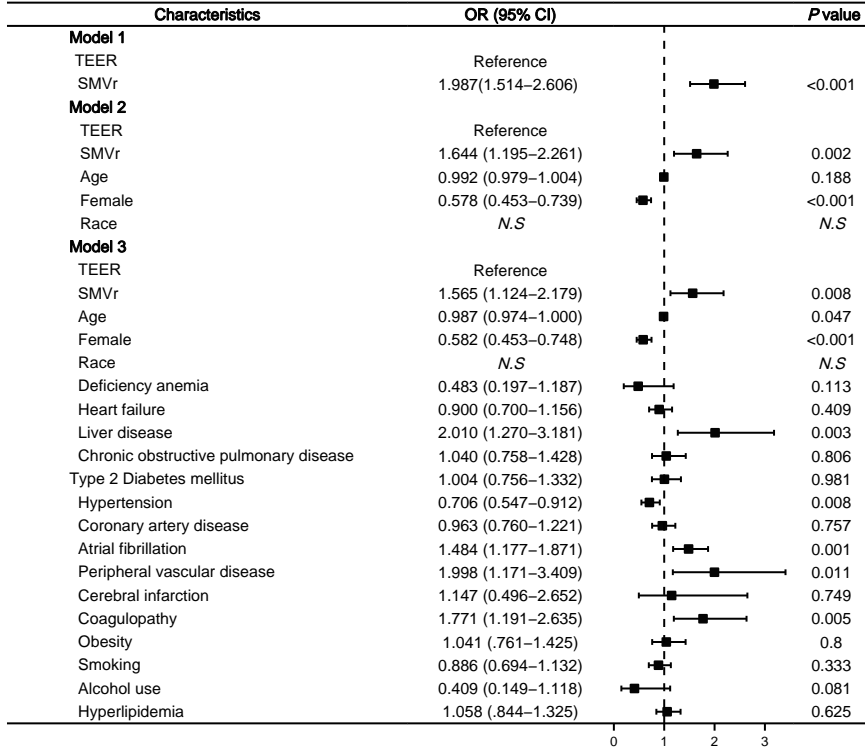

Supplement: Supplementary file 2 — Figure S2. Predictors of postprocedural pericardial complications in mitral valve insufficiency patients undergoing SMVr and TEER. [file CLC-47-e24313-s007.pdf]

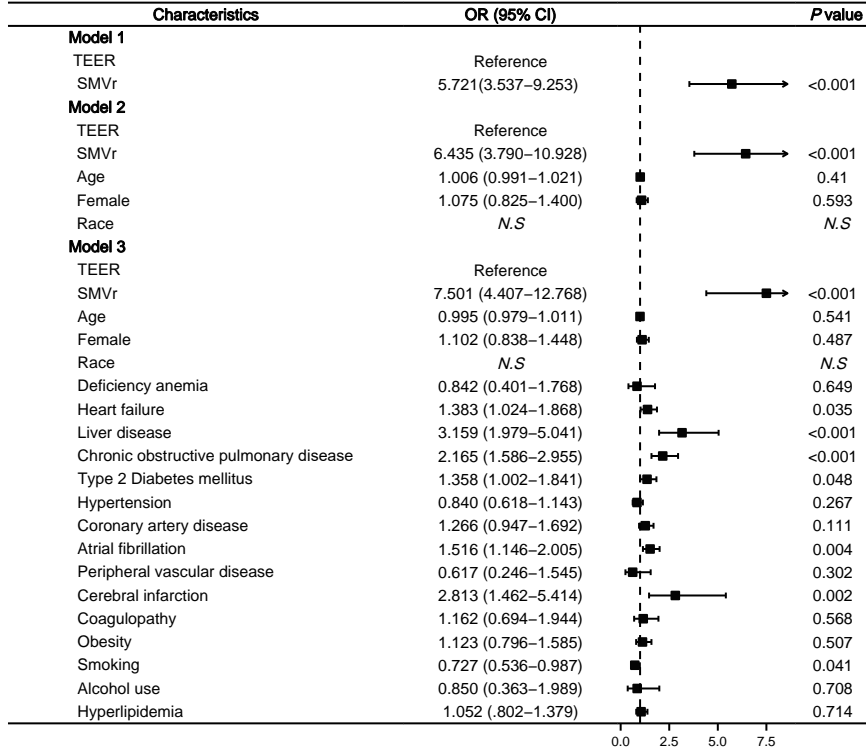

Supplement: Supplementary file 3 — Figure S3. Predictors of postprocedural respiratory failure in mitral valve insufficiency patients undergoing SMVr and TEER. [file CLC-47-e24313-s003.pdf]

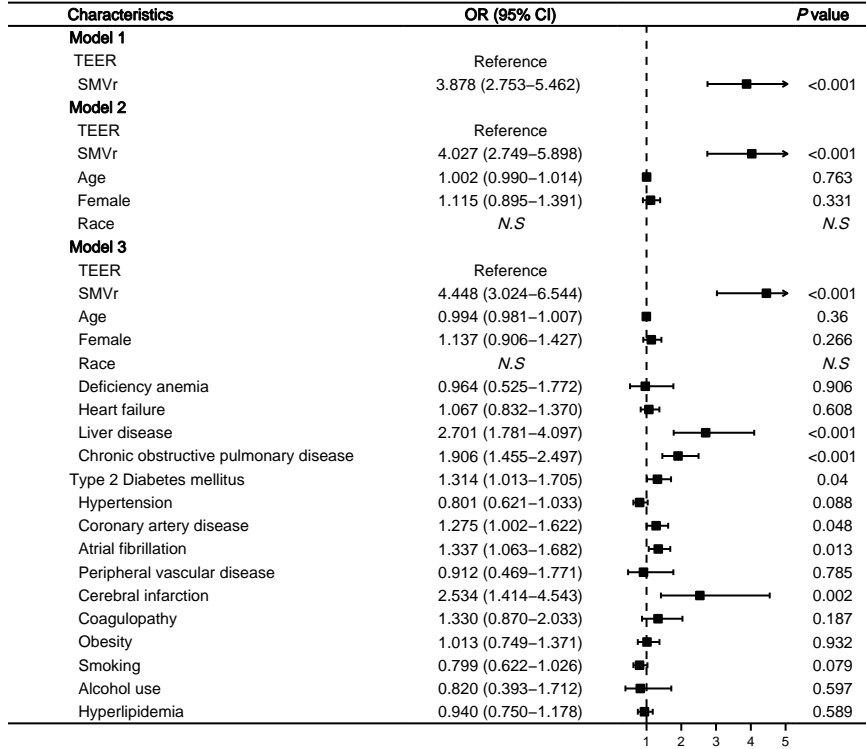

Supplement: Supplementary file 4 — Figure S4. Predictors of Postprocedural Respiratory complications in mitral valve insufficiency patients undergoing SMVr and TEER. [file CLC-47-e24313-s009.pdf]

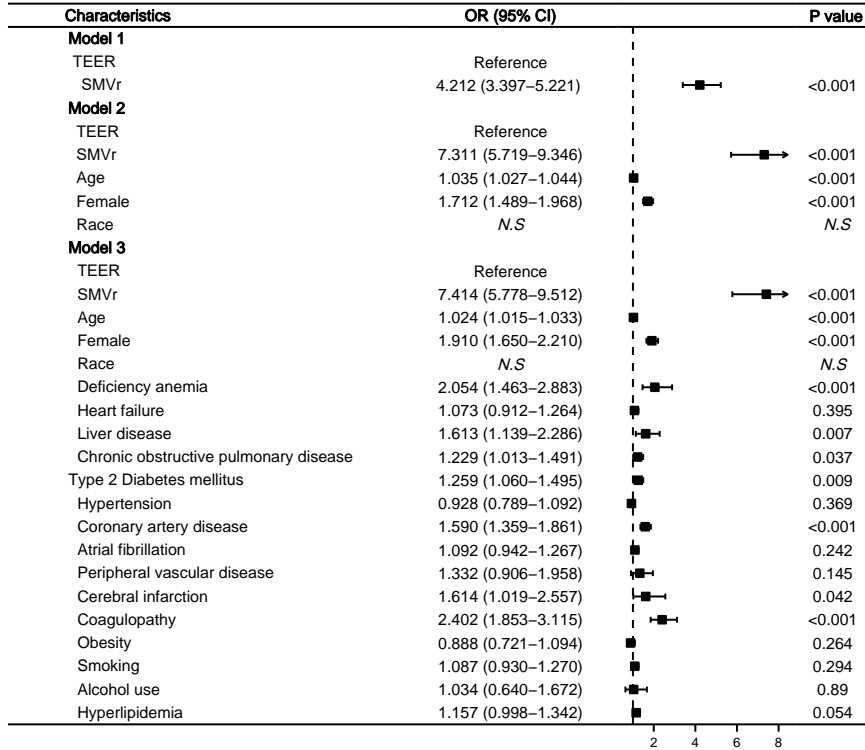

Supplement: Supplementary file 5 — Figure S5. Predictors of Postprocedural Blood transfusion in mitral valve insufficiency patients undergoing SMVr and TEER. [file CLC-47-e24313-s004.pdf]

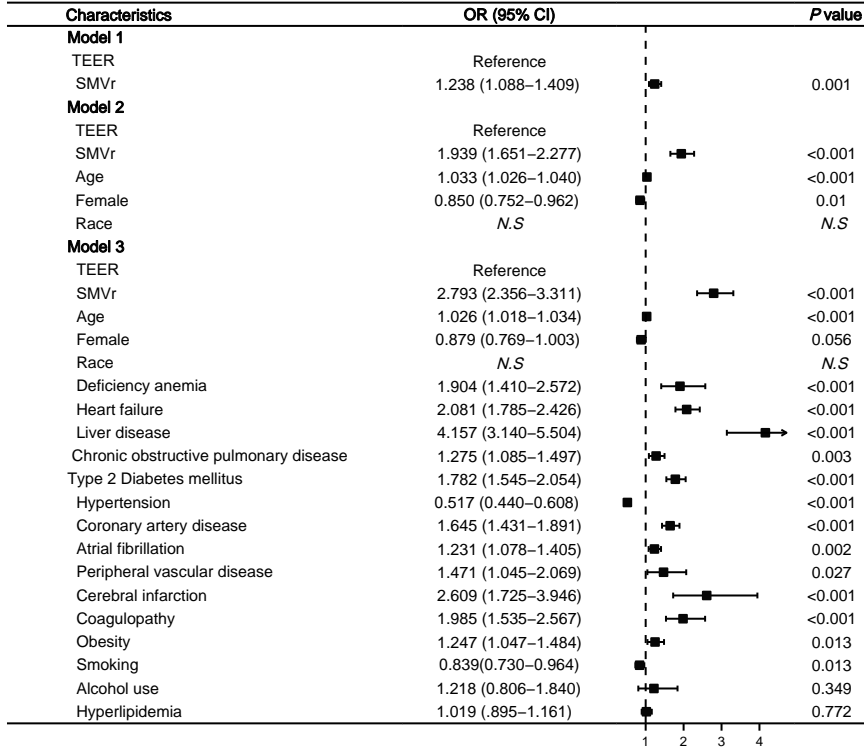

Supplement: Supplementary file 6 — Figure S6. Predictors of Postprocedural Acute kidney injury in mitral valve insufficiency patients undergoing SMVr and TEER. [file CLC-47-e24313-s005.pdf]

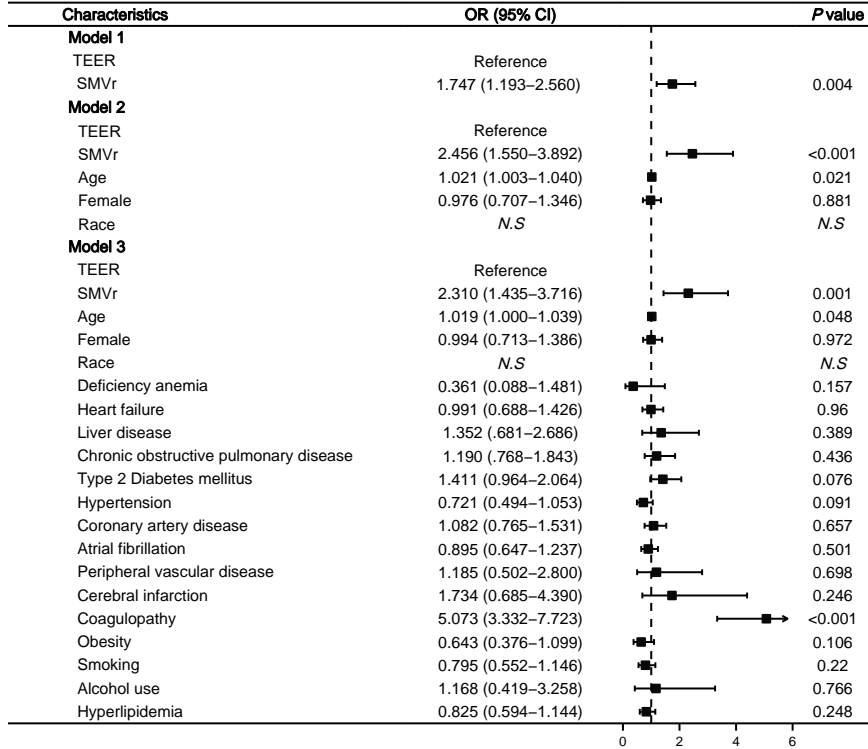

Supplement: Supplementary file 7 — Figure S7. Predictors of Postprocedural Bleeding/hematoma post‐procedure in mitral valve insufficiency patients undergoing SMVr and TEER. [file CLC-47-e24313-s001.pdf]

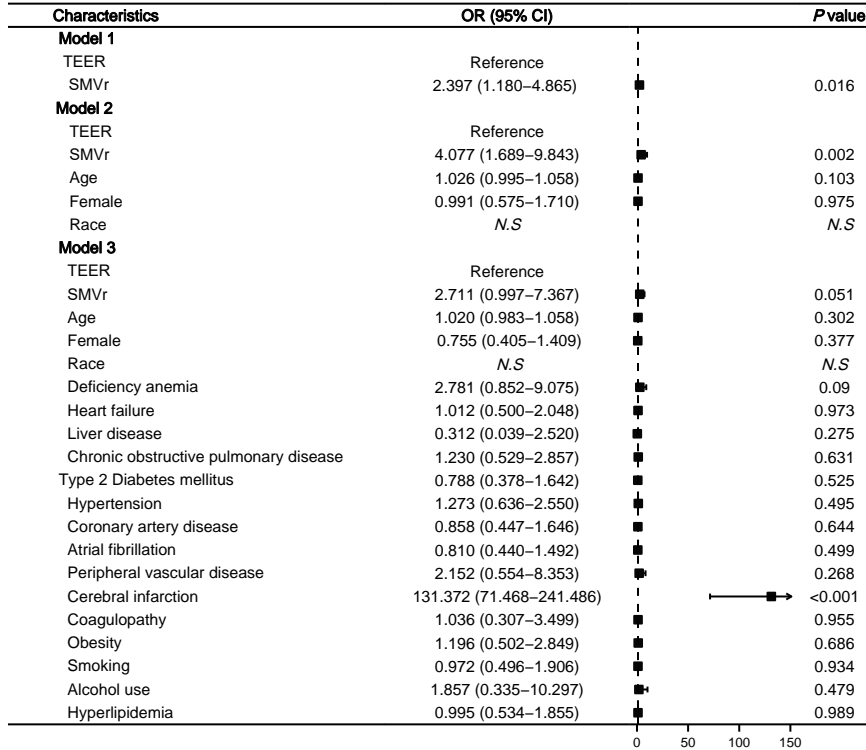

Supplement: Supplementary file 8 — Figure S8. Predictors of Postprocedural cerebrovascular infarction in mitral valve insufficiency patients undergoing SMVr and Mitraclip. [file CLC-47-e24313-s002.pdf]
